# Association between personality traits and food stockpiling for disaster

**DOI:** 10.1371/journal.pone.0259253

**Published:** 2021-12-29

**Authors:** Moeka Harada, Nobuyo Tsuboyama-Kasaoka, Jun Oka, Rie Kobayashi

**Affiliations:** 1 Department of Food and Nutrition, Faculty of Home Economics, Tokyo Kasei University, Tokyo, Japan; 2 Section of Global Disaster Nutrition, International Center for Nutrition and Information, National Institute of Health and Nutrition, National Institutes of Biomedical Innovation, Health and Nutrition, Tokyo, Japan; 3 Department of Rehabilitation, Faculty of Health Sciences, Tokyo Kasei University, Saitama, Japan; Universitat de Valencia, SPAIN

## Abstract

This study investigated the association between personality traits and food stockpiling for disasters in predicted high-risk areas of food shortages due to the Nankai Trough Earthquake. This survey was conducted between December 18 and 20, 2019, using a web-based questionnaire. The participants were 1,200 individuals registered with an online survey company. This study analyzed the association between the Big Five personality traits and food stockpiling status (n = 1192). The Big Five personality traits assess five basic dimensions of personality (i.e., extraversion, conscientiousness, agreeableness, neuroticism, and openness). To measure theses personality traits, we used the Japanese version of the Ten-Item Personality Inventory (TIPI-J). The Mann-Whitney test and a multivariable logistic regression analysis revealed that none of the Big Five personality traits were significantly associated with having or not having stockpile food. However, interestingly, considering the stages of behavior change regarding stockpiling, high extraversion was significantly positively related to initiating stockpiling. Moreover, high neuroticism was significantly positively related to interrupted stockpiling. Therefore, it is crucial to focus on personality traits (especially low extraversion and high neuroticism) to promote food stockpiling for disasters.

## Introduction

Owing to pandemics such as the coronavirus disease (COVID-19), the food supply chain has buckled [1]. Consequently, people’s perceptions of there being a “food shortage” resulted in panic buying [2]. Such panic buying can occur not only during a pandemic but also after natural disasters. Natural disasters disrupt the food supply chain, and people in affected areas face food a scarcity [3], which can further trigger panic buying behavior.

If individuals do not have stockpiles, food assistance from outside the disaster affected area will be required. Consequently, food shortages can be expected even outside the disaster affected areas. In some cases, despite there being enough food available outside the disaster area, it is difficult to deliver to the disaster areas. Post the Great East Japan Earthquake (2011), the Japanese government could not deliver a sufficient amount of food to the affected areas for approximately five days after the disaster [4]. Therefore, if each individual stockpile food in advance, this will help not only the disaster area but also the society outside the disaster area.

Previous studies have reported that stockpiling behaviors, such as panic buying, are associated with personality traits. People with high extraversion and neuroticism are more likely to engage intake panic buying [5]. Personality traits, including extraversion and neuroticism, are categorized as among the Big Five personality traits, a five-factor model that guides various behaviors. The Big Five personality traits comprise extraversion, conscientiousness, agreeableness, neuroticism, and openness [6]. It is believed that personality traits might be related to food stockpiling behavior, similar to panic buying behavior. However, the relationship between personality traits and food stockpiling for disasters remains unknown.

Therefore, the present study aimed to investigate the association between personality traits and food stockpiling for disaster in the areas with a high-risk of food shortage due to the Nankai Trough Earthquake. Additionally, whether personality traits affect individuals’ behavior to start and interrupt stockpiling of food for disasters was investigated.

## Methods

### Design and data collection

Data for this study was obtained through online questionnaire administered to 1,200 Japanese individuals aged ≥20 years, registered with an online survey company (Rakuten Insight, Inc.; a total of 2.2 million registrants). The company ceased recruitment when the total number of participants exceeded the target of 1,200 individuals. The survey was conducted between December 18 and 20, 2019. The company compensated the participants through financial incentives (Rakuten Points).

The study population was recruited from the top five prefectures in Japan that estimated food shortages after the Nankai Trough Earthquake, calculated from the estimated number of evacuees [7] by the Japanese government based on each prefecture’s population [8]. The five prefectures were Kochi, Tokushima, Wakayama, Ehime, and Mie. The target participants were individuals who prepared meals for the family as they were the ones who would presumably also stock food for disaster for the family.

### Questionnaire

A questionnaire comprising 35 items and was self-administered using computers or smartphones. The questionnaire had three parts, as follows:

#### Part 1: Sociodemographic information of individuals

This part included questions regarding participants’ sex, age, employment status, educational background, disaster experience, and community activities (participating or not participating). Additionally, it also included participants’ household income, family composition, and vulnerable people in the family (presence or none).

#### Part 2: Personality traits (Exposure)

We used the Japanese version of the Ten-Item Personality Inventory (TIPI-J) to measure personality traits [9]. The TIPI-J is a measure of the Big Five personality dimensions: extraversion, conscientiousness, agreeableness, neuroticism, and openness [10]. This measure consists of a total of 10 items, two items for each of the Big Five personality traits. Therefore, this measure is easy to answer and is used even in questionnaires with a large number of questions [10, 11]. Tests for validation and reliability of the TIPI-J have been established in Japan [9]. Additionally, it can be used in volunteer panel web surveys [12]. TIPI-J items are rated from 1 (strongly disagree) to 7 (strongly agree).

#### Part 3: Stage of food stockpiling for disaster (Outcome measures)

**Fig 1:**
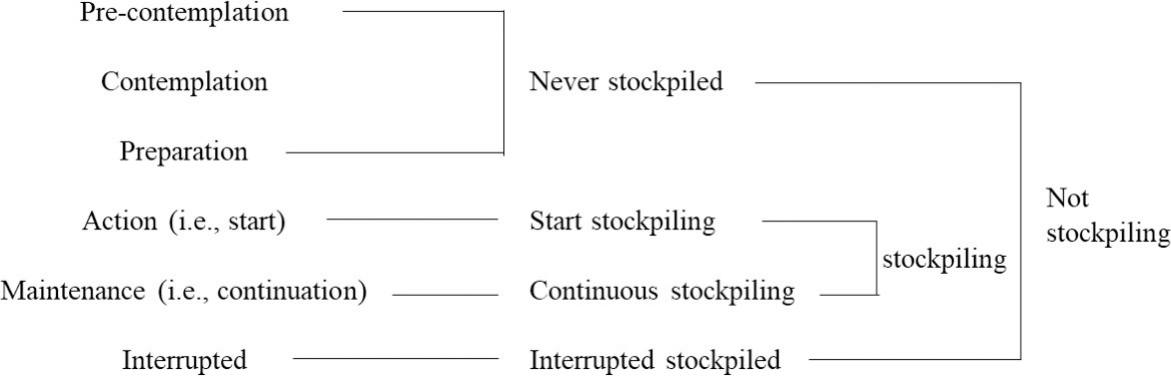
Stage of food stockpiling for disaster [18].

To assess the stage of food stockpiling for disasters, we used the Transtheoretical Model (TTM), which is a model derived from smoking cessation studies [13–15]. TTM is now adapted to the behaviors of eating, exercising, etc. after elucidating its validity [16, 17]. The six items are as follows: 1) Pre-contemplation: not interested in stockpiling of food for disaster, 2) Contemplation: intending to stockpile food for disaster in the next 6 months, 3) Preparation: ready to stockpile food for disaster in the next month, 4) Action: have stockpiled food for disaster but have not replaced it, 5) Maintenance: have stockpiled food for disaster and have replaced it more than once, 6) Interrupted: used to have stockpile before but not now. We combined the stages of “pre-contemplation,” “contemplation,” and “preparation” as “never stockpiled.” The “action” stage was classified as “start stockpiling” and “maintenance” as “continuous stockpiling” (Fig 1).

### Ethics

The study was conducted as per the principles of the Declaration of Helsinki. Ethical approval was granted by the Research Ethics Committee of the Graduate School of Tokyo Kasei University (approval number: R2-3) and the Institutional Ethics Committee of the National Institute of Health and Nutrition (approval number: KENEI127). All participants of this study were registered on an online survey company and provided informed consent prior to participation.

### Statistical analysis

The Mann-Whitney test was performed to compare the Big Five personality scores from TIPI-J. Additionally, logistic regression analysis was performed to evaluate the association between the status of food stockpiling for disaster and personality traits.

First, a logistic regression analysis was conducted using the status of stockpiling disaster food as the dependent variable (reference category: not stockpiled) and the Big Five personality traits as the independent variables. Results are presented as crude or adjusted odds ratios (ORs) with 95% confidence intervals (95% CI). Initially the variables were evaluated using univariable analysis, followed by a multivariable analysis (forced entry method) to adjust for some factors. The adjusted factors, among the participants’ characteristics, were factors associated with food stockpiling in a previous study [18]; including sex (male or female), age (20–34, 35–59 and ≥60 years), educational background (below undergraduate, or above college degree), and community activities (participating or not participating). In total, 1192 responses were recorded into the logistic regression model, after excluding responses related to sex and educational background as “others” (n = 3 and n = 8, respectively).

Second, a sub-analysis on the Big Five personality affecting food stockpiling (“start” or “interrupted”) behavior was conducted. Initially, “start stockpiling” was selected as the dependent variable (reference category: “never stockpiled”). Thereafter, “interrupted stockpiled” was selected (reference category: “continuous stockpiling”). The independent variable was the Big Five personality traits. The adjusted factors were the factors associated with food stockpiling, similar to those in the first logistic regression analysis.

P-values were two-sided, with *p* < 0.05, considered statistically significant. All statistical analyses were performed using the IBM SPSS Statistics version 26.

## Results

In total, 1,200 participants completed the survey, excluding those whose response time was extremely short or who provided an inconsistent answer. Additionally, we excluded participants whose responses to sex and educational background are “others” (n = 3 and n = 8, respectively).

Participant characteristics are presented in Table 1 (n = 1192). A higher number of females than males responded (69.0% vs. 31.0%).

**Table 1:** Participants’ characteristics distinguished by the status of stockpiling disaster food.

		Total	The status of food stockpiling	
		(n = 1192)	Not stockpiling	Stockpiling
			(n = 710)	(n = 482)
		n (%)	n (%)	n (%)
Sex				
	Male	369 (31.0)	249 (35.1)	120 (24.9)
	Female	823 (69.0)	461 (64.9)	362 (75.1)
Age				
	20–34 years	346 (29.0)	225 (31.7)	121 (25.1)
	35–59 years	607 (50.9)	369 (52.0)	238 (49.4)
	≥60 years	239 (20.1)	116 (16.3)	123 (25.5)
Employment status				
	Not employed	406 (34.1)	220 (31.0)	186 (38.6)
	Employed	786 (65.9)	490 (69.0)	296 (61.4)
Educational background				
	Below undergraduate	741 (62.2)	454 (63.9)	287 (59.5)
	Above college degrees	451 (37.8)	256 (36.1)	195 (40.5)
Disaster experience				
	None	979 (82.1)	590 (83.1)	389 (80.7)
	Have experience	213 (17.9)	120 (16.9)	93 (19.3)
Community activities				
	Not participant	818 (68.6)	544 (76.6)	274 (56.8)
	Participating	374 (31.4)	166 (23.4)	208 (43.2)
Prefecture				
	Mie pref.	364 (30.5)	216 (30.4)	148 (30.7)
	Wakayama pref.	219 (18.4)	126 (17.7)	93 (19.3)
	Tokushima pref.	164 (13.8)	93 (13.1)	71 (14.7)
	Ehime pref.	331 (27.8)	205 (28.9)	126 (26.1)
	Kochi pref.	114 (9.6)	70 (9.9)	44 (9.1)
Household income				
	<6 million yen	714 (59.9)	446 (62.8)	268 (55.6)
	≥6 million yen	284 (23.8)	153 (21.5)	131 (27.2)
	unknown	194 (16.3)	111 (15.6)	83 (17.2)
Family composition				
	Single household	356 (29.9)	250 (35.2)	106 (22.0)
	Others	836 (70.1)	460 (64.8)	376 (78.0)
Vulnerable people in family				
	None	877 (73.6)	543 (76.5)	334 (69.3)
	Presence	315 (26.4)	167 (23.5)	148 (30.7)

Excluding participants which responses of sex and educational background are ’the others’ (n = 8).

### Association between the Big Five personality traits and food stockpiling

Table 2 shows the Big Five personality scores based on the status of food stockpiling. No significant difference was found in any Big Five personality scores between the non-stockpiling and stockpiling groups.

**Table 2:** The distribution of Big-Five personality by the status of stockpiling disaster food.

Big Five personality traits from TIPI-J		Total	The status of food stockpiling		*P* value ^†^
			Not stockpiling	Stockpiling	
		(n = 1192)	(n = 710)	(n = 482)	
Extraversion	Mean ± SD	3.83±1.26	3.81±1.25	3.87±1.27	0.481
	Median	4.00	4.00	4.00	
	(25%tile-75%tile)	(3.00–4.50)	(3.00–4.50)	(3.00–4.50)	
Agreeableness	Mean ± SD	4.76±0.96	4.76±0.94	4.77±0.98	0.633
	Median	5.00	5.00	5.00	
	(25%tile-75%tile)	(4.00–5.50)	(4.00–5.50)	(4.00–5.50)	
Conscientiousness	Mean ± SD	3.97±1.13	3.96±1.14	3.98±1.10	0.842
	Median	4.00	4.00	4.00	
	(25%tile-75%tile)	(3.50–4.50)	(3.50–4.50)	(3.50–4.50)	
Neuroticism	Mean ± SD	4.09±1.15	4.11±1.13	4.07±1.17	0.565
	Median	4.00	4.00	4.00	
	(25%tile-75%tile)	(3.50–5.00)	(3.50–5.00)	(3.50–5.00)	
Openness	Mean ± SD	3.90±1.04	3.90±1.05	3.89±1.04	0.732
	Median	4.00	4.00	4.00	
	(25%tile-75%tile)	(3.50–4.50)	(3.50–4.50)	(3.38–4.50)	

Excluding participants which responses of sex and educational background are ’the others’ (n = 8).

† Mann-Whitney test was performed for each Big-Five personality of TIPI-J.

As shown in Table 3, logistic regression analysis reported that none of the Big Five personality traits were significantly associated with food stockpiling.

**Table 3:** Logistic regression results for association between the Big Five personality traits and food stockpiling for disaster (“stockpiling” vs “not stockpiling”) (n = 1192) ^†^.

Big Five personality traits from TIPI-J	Crude	Adjusted^§^
	OR (95% CI)	OR (95% CI)
Extraversion	1.04 (0.95–1.14)	1.06 (0.96–1.16)
Agreeableness	1.01 (0.90–1.14)	0.99 (0.87–1.12)
Conscientiousness	1.02 (0.92–1.13)	1.01 (0.90–1.12)
Neuroticism	0.97 (0.88–1.08)	0.99 (0.90–1.10)
Openness	0.99 (0.88–1.10)	1.00 (0.89–1.12)

† Excluding participants which responses of sex and educational background are ’the others’ (n = 8).

§ Sex, age, educational background, and community activities were adjusted in the adjusted model.

*** *p* < 0.001, ** *p* < 0.01, * *p* < 0.05

OR: Odds Ratio, CI: Confidence intervals

### Association between the Big Five personality traits and food stockpiling stage (“start” or “interrupted”)

Table 4 shows the Big Five personality scores from TIPI-J by the food stockpiling stage. The extraversion score for the “start stockpiling” group was significantly higher than that for the “never stockpiled” group (4.01 ± 1.22 vs. 3.80 ± 1.25, respectively, *p* < 0.05). The neuroticism score for the “interrupted stockpiled” group was significantly higher than that for the “continuous stockpiling” group (4.32 ± 1.07 vs. 4.05 ± 1.19, respectively, *p* < 0.01).

**Table 4:** The distribution of the Big Five personality traits by food stockpiling stage.

Big Five personality traits from TIPI-J		The stage of food stockpiling for disaster					
		"Never" vs. "Start"			"Continuous" vs. "Interrupted"		
		Never	Start	*P* value ^†^	Continuous	Interrupted	*P* value ^†^
		(n = 539)	(n = 182)		(n = 300)	(n = 171)	
Extraversion	Mean ± SD	3.80 ± 1.25	4.01 ± 1.22	< 0.05	3.79 ± 1.30	3.83 ± 1.27	
	Median	4.00	4.00		4.00	4.00	
	(25%tile-75%tile)	3.00–4.50	3.00–5.00		3.00–4.50	3.00–4.50	
Agreeableness	Mean ± SD	4.75 ± 0.94	4.71 ± 1.03		4.80 ± 0.95	4.79 ± 0.94	
	Median	5.00	5.00		5.00	5.00	
	(25%tile-75%tile)	4.00–5.50	4.00–5.50		4.00–5.50	4.00–5.50	
Conscientiousness	Mean ± SD	3.98 ± 1.13	3.95 ± 1.07		4.00 ± 1.11	3.90 ± 1.20	
	Median	4.00	4.00		4.00	4.00	
	(25%tile-75%tile)	3.50–4.50	3.00–4.50		3.50–4.50	3.00–4.50	
Neuroticism	Mean ± SD	4.04 ± 1.14	4.10 ± 1.15		4.05 ± 1.19	4.32 ± 1.07	< 0.01
	Median	4.00	4.00		4.00	4.00	
	(25%tile-75%tile)	3.50–4.50	3.50–4.63		3.50–5.00	3.50–5.00	
Openness	Mean ± SD	3.92 ± 1.05	3.86 ± 1.01		3.91 ± 1.05	3.85 ± 1.04	
	Median	4.00	4.00		4.00	4.00	
	(25%tile-75%tile)	3.50–4.50	3.00–4.50		3.50–4.50	3.00–4.50	

Excluding participants which responses of sex and educational background are ’the others’ (n = 8).

† Mann-Whitney test was performed for each Big-Five personality of TIPI-J.

As shown in Table 5, individuals with a high extraversion score were 1.17 times more likely to start stockpiling food (adjusted OR: 1.17, 95% CI: 1.02–1.34, *p* for trend < 0.05). The other personality score and “start stockpiling” (reference category: “never stockpiled”) were not significantly associated.

**Table 5:** Logistic regression results for association between Big Five personality traits and food stockpiling stage; “start stockpiling” vs “never stockpiled” (n = 721) ^†^.

Big Five personality traits from TIPI-J	Crude	Adjusted ^‡^
	OR (95% CI)	OR (95% CI)
Extraversion	1.15 (1.00–1.31) *	1.17 (1.02–1.34) *
Agreeableness	0.96 (0.81–1.14)	0.91 (0.78–1.11)
Conscientiousness	0.98 (0.84–1.14)	0.97 (0.83–1.13)
Neuroticism	1.05 (0.91–1.22)	1.07 (0.92–1.24)
Openness	0.95 (0.81–1.12)	0.96 (0.81–1.13)

Excluding participants which responses of sex and educational background are ’the others’ (n = 8).

† Reference category of the dependent variable is “Never stockpiled.”

‡ Sex, age, educational background, and community activities were adjusted in the adjusted model.

*** *p* < 0.001, ** *p* < 0.01,

* *p* < 0.05 (*p* for trend)

OR: Odds Ratio, CI: Confidence intervals

Table 6 shows that individuals with a high neuroticism score were 1.20 times more likely to have interrupted stockpiling of food (adjusted OR: 1.20, 95% CI: 1.01–1.43, *p* for trend < 0.05). The other personality score was not significantly associated with “interrupted stockpiled” (reference category: “continuous stockpiling”).

**Table 6:** Logistic regression results for association between Big Five personality traits and food stockpiling stage; “interrupted stockpiled” vs “continuous stockpiling” (n = 471) ^†^.

Big Five personality traits from TIPI-J	Crude	Adjusted ^‡^
	OR (95% CI)	OR (95% CI)
Extraversion	1.02 (0.88–1.19)	1.03 (0.89–1.20)
Agreeableness	0.99 (0.81–1.20)	1.00 (0.82–1.22)
Conscientiousness	0.92 (0.78–1.09)	0.93 (0.78–1.10)
Neuroticism	1.23 (1.04–1.45) *	1.20 (1.01–1.43) *
Openness	0.95 (0.80–1.14)	0.95 (0.79–1.14)

Excluding participants which responses of sex and educational background are ’the others’ (n = 8).

† Reference category of the dependent variable is “Continue stockpiling.”

‡ Sex, age, educational background, and community activities were adjusted in the adjusted model.

*** *p* < 0.001, ** *p* < 0.01,

* *p* < 0.05 (*p* for trend)

OR: Odds Ratio, CI: Confidence intervals

## Discussion

This study investigated the association between personality traits and food stockpiling for disasters at home using an online survey. The results showed that the Big Five personality traits were not significantly associated with having or not having stockpile food. However, a detailed analysis of the stockpiling behavior stage based on TTM showed that high extraversion was significantly positively associated with starting to stockpile. Moreover, high neuroticism was significantly positively associated with the interrupted stockpiling of food.

### Association between the Big Five personality traits and food stockpiling

An unexpected result was that no personality trait was significantly associated with stockpiling or not stockpiling of food. Previous studies have compared groups with no stockpiles to groups that have stockpiles [19–23]. However, it is considered that groups with no stockpile include two behavior stages: those who have never stockpiled and those who have been interrupted (used to have stockpile before but not now). Interestingly, in our study, although there were no significant personality traits between having and not having to stockpile, personality traits were observed for start and interrupted stockpiling of food. In the TTM, the appropriate approach differs depending on the stage of behavior change [13–15]. Therefore, our results showed that it is important to investigate the risk factors for no stockpiles considering the stages of behavior change during stockpiles.

### Association between big-five personality and food stockpiling stage (“start” or “interrupted”)

Considering the stage of food stockpiling, it was found that high extraversion was associated with start stockpiling. The extraverted individual is talkative and energetic, and prefers communication with the outside [24]. In a previous study that examined the association between personality and eating styles and food choices, it was shown that people with high extraversion often choose external eating [25]. Hence, it is considered that people with high extraversion prefer to communicate with the outside when choosing food. In other words, they take in more of the opinions from others and choose food. Additionally, participation in community activities leads to stockpiling emergency kits [26]. Therefore, regarding food stockpiling, it may be that extroversion, which is characteristic of talkative people [24], influenced participation in community activities and led to the start of stockpiling food for disasters.

To promote the start of food stockpiling, it was considered effective to approach people with low extroversion. They tend to think things over carefully for themselves without communication with others [24]. They also have a personality trait in which evaluations and praise from others do not lead to actions [27]. Therefore, it would be needed an approach that focuses on their own interests rather than the evaluation of others. For example, the following approach may be effective: education and provision of knowledge that food stockpiling is an important activity important for survival several days after a disaster occurs, product development of stockpiled food that makes them want to collect, etc.

In contrast, interrupted stockpiling is associated with neuroticism. A previous study showed that people with high neuroticism experience difficulty in continuing favorable food choices [25]. Additionally, a study among Japanese people indicated that individuals with high neuroticism find it difficult to exercise [28]. Because they have a personality trait that makes it difficult to continue behavior due to emotional instability without being able to control emotions [29, 30]. Therefore, this personality is also considered to be associated with the difficulty of continuing food stockpiling for disasters (i.e., “interrupted stockpiled”).

To promote people with high neuroticism to continue food stockpiling, they need a way that is independent of their emotions; that is, they can continue automatically. For example, it may be effective to adopt a system such as a subscription that can be automatically renewed once started.

Additionally, introducing a system of subscriptions may also help avoid food waste problems. While food stockpiling is useful in the event of a disaster, it is also true that food stockpiling that is never used may contribute to food waste [2]. By automatically delivering new food before the expiration date of the stockpiled food, it is possible to remember the date of the food. Therefore, subscriptions may also solve the problem of food waste.

### Limitations of this study

This study has the following limitations. First, the study participants were recruited by monitors of an online survey company, which was conducted only in a specific area. Since the study area was relatively rural in Japan, it might show different trends in the urban areas. Accordingly, the generalization of results may be limited because the selection bias cannot be denied. Second, the questionnaire (excluding TIPI-J [12]) did not examine the validity of online surveys, excluding TIPI-J. Although the questionnaire included items based on TTM, further studies are required to verify whether this modification is appropriate. Third, the study participants were limited to those who prepared meals for their families. As a result, many of the respondents were women (69.0%). Japanese women spend more time doing housework than men. Generally, it is considered that food for stockpiling is more likely to be purchased by people who prepare meals in the family compared to the purchase of emergency kits. The Japanese national survey reported that 68.0% of the respondents were women in the responses of those who were in charge of food stockpiling. However, further consideration is required to determine whether the selection of survey targets is appropriate because risk factors could change depending on who in the family is stockpiling. Fourth, since this study had a cross-sectional design, we were unable to confirm the presence of causal relationships. In contrast, there were two advantages to using an online survey: 1) There was no bias due to the influence of the researcher, and 2) There was no substitute response (it is highly possible that we could certainly obtain responses from the study target, which referred to people who prepared meals for the family).

## Conclusion

None of the Big Five personality traits showed any significant association with having or not having food stockpile in the high-risk of food shortage areas due to the Nankai Trough Earthquake. However, considering the stages of behavior change in stockpiling, high extraversion was significantly positively associated with the start of stockpiling. Furthermore, high neuroticism was significantly positively associated with interrupted stockpiling. Therefore, it may be useful to approach those with “low extroversion” and “high neuroticism” to promote food stockpiling for disasters.

## Supporting information

S1 File Questionnaire. (DOCX)
