# Supplementary material for: Association between personality traits and food stockpiling for disaster
Source: PLoS One. 2021 Dec 29;16(12):e0259253. doi: 10.1371/journal.pone.0259253 (PMC8716029; doi:10.1371/journal.pone.0259253)
Supplement: S1 File — (DOCX) [file pone.0259253.s001.docx]

**Questionnaire** (The items used in this study are shown below.)

SC1. Who in your family mainly prepares meals?

1. My self
2. Someone other than myself
3. Do not eat at home

SC2. Please mention your age.

__ years old

SC3. Please specify your sex.

1. Male
2. Female
3. Others

Q1. Please provide your employment status.

1. Employed (including a part-time job)
2. Unemployed (including househusband/housewife, job seeker, and maternity/childcare leave)

Q2. Please mention your educational background.

1. Junior high school
2. High school
3. Professional and vocational junior college
4. Junior college/College of technology
5. University
6. Graduate school
7. Others

Q3. (Not used in this study)

Q4. Have you ever encountered a disaster that made you feel you were in physical danger?

1. Yes
2. No

Q5. (Not used in this study)

Q6. (Not used in this study)

Q7. (Not used in this study)

Q8. Here are a number of personality traits that may or may not apply to you. Please write a number next to each statement to indicate the extent to which you agree or disagree with that statement. You should rate the extent to which the pair of traits applies to you, even if one characteristic applies more strongly than the other.

1 = Disagree strongly, 2 = Disagree moderately, 3 = Disagree a little, 4 = Neither agree nor disagree, 5 = Agree a little, 6 = Agree moderately, 7 = Agree strongly

I see myself as:

1. Extraverted, enthusiastic.
2. Critical, quarrelsome.
3. Dependable, self-disciplined.
4. Anxious, easily upset.
5. Open to new experiences, complex.
6. Reserved, quiet.
7. Sympathetic, warm.
8. Disorganized, careless.
9. Calm, emotionally stable.
10. Conventional, uncreative.

Q9. (Not used in this study)

**■ Please state your habits.**

Q10. How often have you participated in community activities in the past year?

1. Regularly
2. Sometimes
3. Not participated

Q11-22. (Not used in this study)

**■ Please tell us about your family you live with.**

Q23. Please tell us about your family composition.

1. Single household (living by myself)
2. Household with married couple only
3. Household with only married couple and unmarried children
4. Household with only single parents and unmarried children
5. Three-generation household
6. Others

Q24. Please select the closest of the following about your household income over the past year.

1. <2 million yen
2. 2–6 million yen
3. ≥6 million yen

Q25. Do you have vulnerable people in a disaster in your family? Please select all that apply.

1. None
2. Infant
3. Child
4. Pregnant
5. Disabled person
6. Elderly person
7. Person with a chronic disease
8. Person with any food allergies
9. Person who do not speak Japanese
10. Person with pressure ulcers
11. Others

Q26. (Not used in this study)

Q27. Please select the item that applies to your status of food stockpiling for disaster at home.

1. I am not interested in stockpiling of food for disaster.
2. I am intending to stockpile food for disaster in the next 6 months, but not within the next month.
3. I am ready to stockpile food for disaster in the next month.
4. I have stockpiled food for disaster but have not replaced it.
5. I have stockpiled food for disaster and have replaced it more than once.
6. I used to have stockpile before but not now.

Q28-32. (Not used in this study)
